# Hydropower's Biogenic Carbon Footprint

**DOI:** 10.1371/journal.pone.0161947

**Published:** 2016-09-14

**Authors:** Laura Scherer, Stephan Pfister

**Affiliations:** Institute of Environmental Engineering, ETH Zurich, Zurich, Switzerland; Potsdam Institute for Climate Impact Research, GERMANY

## Abstract

Global warming is accelerating and the world urgently needs a shift to clean and renewable energy. Hydropower is currently the largest renewable source of electricity, but its contribution to climate change mitigation is not yet fully understood. Hydroelectric reservoirs are a source of biogenic greenhouse gases and in individual cases can reach the same emission rates as thermal power plants. Little is known about the severity of their emissions at the global scale. Here we show that the carbon footprint of hydropower is far higher than previously assumed, with a global average of 173 kg CO_2_ and 2.95 kg CH_4_ emitted per MWh of electricity produced. This results in a combined average carbon footprint of 273 kg CO_2e_/MWh when using the global warming potential over a time horizon of 100 years (GWP100). Nonetheless, this is still below that of fossil energy sources without the use of carbon capture and sequestration technologies. We identified the dams most promising for capturing methane for use as alternative energy source. The spread among the ~1500 hydropower plants analysed in this study is large and highlights the importance of case-by-case examinations.

## Introduction

The annual emission rates of carbon dioxide (CO_2_) and methane (CH_4_), and the consequent global temperature increase, are accelerating rapidly [1]. To meet the target of limiting the average temperature increase to 2°C, emission reductions are urgently needed. Even in the case that emissions are halved in 2050 compared to 1990, there is still a 29% chance that the target is missed [2]. A portfolio of strategies, coined the stabilization wedges, is available in order to limit emissions using existing technologies. These strategies include energy efficiency increases, carbon capture and storage (CSS), alternative (nuclear and renewable) energy sources and forest and agricultural conservation [3]. Among these, hydropower is considered a low-carbon technology helping to mitigate climate change [4]. However, it was ascertained that the carbon footprint of some hydropower plants is even larger than that of thermal power plants [5]. Emissions are particularly high in tropical regions [6,7] and at high area-to-electricity ratios [5,8].

The emissions from hydroelectric reservoirs arise from the decomposition of organic matter that was either flooded during reservoir construction, transferred to the reservoir by river runoff, grown in the reservoir such as by algal production [6], stems from dead trees protruding from the water [9], or was grown in newly created marshes in the drawdown area [10]. Besides reservoirs, also other ecosystems influence the greenhouse gas (GHG) fluxes. While rivers and lakes emit GHGs, forests, peatlands and wetlands rather bind them. The real impact of the reservoir is therefore the difference between GHG emissions before and after flooding. The net emissions can either be larger than the gross emissions when carbon sinks are lost or smaller when emissions have already occurred [6,11]. In the investigated cases, the sink [6] or source [11] prior to flooding is small compared to the emissions afterwards so that, when also taking into account the uncertainties, it can be considered as carbon-neutral in non-tropical [11] as well as tropical regions [12].

Barros et al. [7] compiled data of about 100 CO_2_ and CH_4_ emission measurements, as well as some reservoir and site characteristics, from published literature. Based on this data, he estimated total emissions of hydroelectric reservoirs and analysed the relationship with explanatory variables. Hertwich [8] supplemented the dataset with electricity information and an additional predictor variable to carry out similar analyses. This study sets up a new statistical model, including additional environmental variables, and applies this model to a global dataset of ~1500 plants (~43% of global hydropower generation) in order to get a better estimate of hydropower’s total climate change impact and its range among individual plants.

## Materials and Methods

### Data collection

We analysed the carbon footprints of 1473 hydroelectric reservoirs across 104 countries, under the assumption that the emissions prior to flooding are negligible and do not significantly change the net footprint [11]. Reservoir locations, its purpose and key characteristics were given in the Global Reservoir and Dam (GRanD) database [13]. This information was linked to annual electricity generation provided by the CARMA database [14]. The analyses represent the year 2009.

CO_2_ and CH_4_ emissions, electricity information and site characteristics as potential predictors of about 100 hydroelectric reservoirs were compiled by Barros et al. [7] and Hertwich [8] (S1 File). This data serves as a training dataset for statistical modelling from which we estimate the emissions of the above mentioned 1473 reservoirs.

We tested various variables as predictors including area, area-to-electricity ratio, age (derived from the year when the dam construction was completed), volume, volume-to-area ratio (as proxy for depth), mean, minimum and maximum temperature [15], net primary productivity [16], topsoil organic carbon content [17] and erosion rate [18] as well as their logarithms. All predictors are available in global datasets; however, the dissolved organic carbon (DOC) used as a predictor by Barros et al. [7] was not available as a global dataset and was therefore excluded from our analysis. The erosion rate serves as a proxy indicator thereof. If the year of dam completion was missing, various online sources were consulted including the AQUASTAT dam database [19].

### Generalized linear modelling

We estimated carbon emissions per energy unit (kg/MWh, Model 1) and as areal fluxes (mg C m^-2^ d^-1^, Model 2) using generalized linear models (GLMs) and combined the two models by averaging (Model A). A Gaussian distribution was assumed for CO_2_ and a logarithmic distribution for CH_4_. In a first step of selecting predictors, explanatory variables were excluded if they correlated with other predictors (correlation coefficient R ≥ 0.5), but have a weaker effect on the model output than the alternative predictor. In a second step, we applied multi-model inference [20] during which competing models with different predictor combinations and numbers of predictors were compared. The analysis was carried out in R using the package MuMIn [21] in which models are compared based on Akaike weights, a transformation of Akaike’s information criterion (AIC) [22]. The final models were evaluated by multiple criteria in order to ensure a more robust assessment [23]. The criteria used were deviance explained (DEX), also called adjusted R^2^, AIC, the percent bias (PBIAS), the normalized root mean square error (NRMSE) and the normalized mean absolute error (NMAE). The DEX and AIC reward a good model fit, but penalize a large number of predictors, which potentially leads to overfitting. Similar to the coefficient of determination (R^2^), a higher value of DEX closer to 1 indicates superior model performance. By contrast, a smaller value of AIC implies better model performance. The remaining criteria only evaluate the performance. Previous models using the same training dataset [7,8] were rebuilt and the performance was compared to our own efforts.

The measurements in the training dataset are likely to contain errors: 1) for CO_2_ due to negligence of carbon burial and 2) for CH_4_ due to negligence of methane ebullition (bubbling) or sampling errors. Therefore, correction factors were derived and it was assumed that 1) the predicted carbon footprints should be 13% lower and these excess emissions should be deducted from CO_2_, and 2) predicted CH_4_ emissions are underestimated and should be increased by a factor of 1.4 (Model A_C_, S2 File).

### Global estimates

The GLMs were subsequently applied to the global dataset of 1473 hydroelectric reservoirs for which the predictors were extracted from the same global datasets as the training dataset. CO_2_ and CH_4_ emissions were aggregated to carbon dioxide equivalents (CO_2e_) by assuming a global warming potential of 34 kg CO_2e_/kg CH_4_ over a time horizon of 100 years (GWP100) and for sensitivity analyses also assuming a global warming potential of 86 kg CO_2e_/kg CH_4_ over a time horizon of 20 years (GWP20) [24]. Although the global warming potential of biogenic CO_2_ can differ from fossil CO_2_, they were assumed to be equal, as the flooded biomass is not replanted and therefore no significant removal of emissions during regrowth can take place [25]. Potential sequestration of CO_2_ in algae and fish biomass of dams is supposed to be marginal compared to terrestrial vegetation (S3 File), but might be evaluated more specifically in future research. First, we calculated production-weighted average emissions per energy unit and then we derived total emissions by multiplying these with the global hydroelectricity generation in 2009, which amounted to 3551 TWh [26]. In addition, we calculated the median and maximum of the reservoirs’ carbon emissions. Plants with a high potential for energy recovery from methane emissions (Model A_C_) were defined as plants that produce at least 10 kg CH_4_/MWh and contribute to at least 50% to the carbon footprint. All results are presented in more detail in the supporting information (S1 Table).

### Allocation

Since reservoirs often fulfil multiple purposes, the environmental impacts should not solely be attributed to hydropower. Instead, the responsibility should be shared among the purposes of the reservoir. Therefore, allocation factors (f_A_) were applied. These factors are based on the ranking of hydropower among all of the n purposes that the reservoir fulfils [27]:

$$f_{A}=\frac{n+1-ranking}{\sum_{i=1}^{n}i}$$ (1)

## Results

### Model set-up and evaluation

We set up generalized linear models (GLMs) for estimating CO_2_ and CH_4_ emissions based on a training dataset of ~100 hydroelectric reservoirs [7,8]. The best model (Model 1) for CO_2_ emissions per energy unit (kg CO_2_/MWh) includes only two predictors: area-to-electricity ratio (ATE, km^2^/GWh) and area (A, km^2^), while the best model for CH_4_ emissions (kg CH_4_/MWh) used age (AGE, years), ATE and maximum temperature (TMX, °C):

$${CO}_{2}=–169.73+241.86\;∙\;ATE+120.34\;∙\;ln(A)$$ (2)

$$ln({CH}_{4})=–9.81–0.75\;∙\;ln(AGE)+1.18\;∙\;ln(ATE)+4.50\;∙\;ln(TMX)$$ (3)

As an alternative, we calculate areal fluxes (Model 2) of GHG emissions (mg C m^-2^ d^-1^), which are independent of the energy production and can also be applied to reservoirs with purposes other than hydropower. In the optimal GLM, the model includes the predictors AGE and the erosion rate (ERR, t ha^-1^ a^-1^) for CO_2_, and additionally A and TMX for CH_4_:

$${CO}_{2}=494.46–4.07\;∙\;AGE+8.09\;∙\;ERR$$ (4)

$$ln({CH}_{4})=–12.84–0.03\;∙\;AGE+0.21\;∙\;ln(A)–0.01\;∙\;ERR+4.88\;∙\;ln(TMX)$$ (5)

As already pointed out by Hertwich [8], the ATE is the single most important predictor for emissions per energy unit. Besides river discharge, the hydroelectricity output is largely determined by the dam height [28], which, in turn, depends strongly on the topography. Mountainous regions with narrow deep river channels require less land and are therefore preferable sites for reservoirs [29,30].

TMX can be interpreted as a proxy indicator to distinguish tropical from non-tropical reservoirs. The higher the TMX, the more likely the reservoir is situated in a tropical region and therefore the higher the CH_4_emissions. This finding is in accordance to previous studies that reported greater emissions, especially of CH_4_, from tropical reservoirs [6,7].

While CH_4_ emissions decline with reservoir age, no significant relationship was found between CO_2_ emissions and age when setting up the model for GHG emissions per MWh. However, Barros et al. [7] and Hertwich [8] included an inverse relationship between age and both types of emissions and it has been included in our alternative approach, with higher weight for CO_2_ (Eq 4). Some previous studies confirmed the decrease with age for CO_2_ [6,7,11] and CH_4_ [7]. By contrast, other studies could not ascertain a relationship between CH_4_ and age [6] or even found an increasing trend during initial years with a levelling-off at a later stage [11]. It has to be noted that our results reflect the analysis across hydropower plants as a result of the training dataset and is not resulting from time series analyses of specific power plants. Since the production of CH_4_ strongly depends on the flooded ecosystem type and measurement procedures can deviate (especially with regards to the inclusion of bubbling in CH_4_ measurements) [6], it is difficult to predict the relationship of CH_4_ emissions to age, and this might explain the differences among studies.

The erosion rate was shown to be a significant predictor for modelling areal fluxes of CO_2_. It is a proxy indicator for biomass continually transported by the river discharge to the reservoir as opposed to the biomass from the initial flooding. How erosion influences CH_4_ fluxes is unclear and it remains to be investigated if the small weight of the predictor is a statistical artefact or if there is a weak causal relationship on a global level.

The model of emissions per energy unit better predicts the measurements than the model of areal fluxes. The same could be observed by comparing the model performance of Barros et al. [7] for areal fluxes and of Hertwich [8] for emissions per energy unit. As indicated by multiple performance criteria, the newly developed models outperform those of Barros et al. [7] and Hertwich [8] with the exception of the model predicting areal CO_2_ fluxes, which performs comparably to the previous model (Table 1). However, by including the ERR and TMX, the models take into account additional environmental conditions relevant for the generation of greenhouse gases, whereas the latitude used as predictor by Barros et al. [7] is not a physical parameter in itself, but might represent the temperature gradient.

**Table 1:** Model comparison based on the deviance explained (DEX), the Akaike information criterion (AIC), the percent bias (PBIAS), the normalized root mean square error (NRMSE) and the normalized mean absolute error (NMAE).

Per energy unit (Model 1)	**CO**_**2**_		**CH**_**4**_	
	This study	Hertwich [8]	This study	Hertwich [8]
DEX	0.940	0.938 (0.944)^a^	0.837	0.715 (0.786)^a^
AIC	1675	1680	313	359
PBIAS^b^	0.0	0.0	-28.9	102.5
NRMSE^b^	0.243	0.247	1.114	3.142
NMAE^b^	0.602	0.623	1.208	2.727
Areal fluxes (Model 2)	This study	Barros et al. [7]	This study	Barros et al. [7]
DEX	0.324 (0.311)^c^	0.276 (0.40)^d^	0.670 (0.785)^c^	0.444 (0.53)^d^
AIC	2127^e^ (154)^c^	159	288 (188)^c^	239
PBIAS^b^	22.3 (-25.7)^c^	-16.4	-50.3 (-44.2)^c^	-22.0
NRMSE^b^	0.849 (0.928)^c^	0.902	1.051 (0.896)^c^	1.006
NMAE^b^	0.712 (0.546) ^c^	0.425	1.114 (0.787)^c^	0.983

^a^ The DEXs reported by Hertwich [8] are provided in parentheses. The difference stems from replacing the net primary productivity (NPP) reported in Hertwich [8] with values extracted from a global dataset [16], in order to reduce missing values. The values in parentheses could be reconstructed.

^b^ All model outputs were transformed to linear emissions per energy unit before evaluating the model performance.

^c^ Values in parentheses represent model performance of a subset of the training dataset to make the two models comparable.

^d^ The DEXs reported by Barros et al. [7] are provided in parentheses. These values could not be reconstructed. For our own evaluation of their model, we did not add 400 kg CO_2_/MWh to the CO_2_ emissions (as they did in order to avoid negative emissions) and we excluded eight values that produced missing values in our own model.

^e^ Our CO_2_ model estimated untransformed CO_2_ emissions, whereas an alternative model was set up to estimate logarithmic emissions to make it comparable to the model by Barros et al. [7]. Since the AIC also depends on the magnitude of the model output, models estimating untransformed and transformed outputs cannot be directly compared.

### Model application to a global dataset

**Fig 1:**
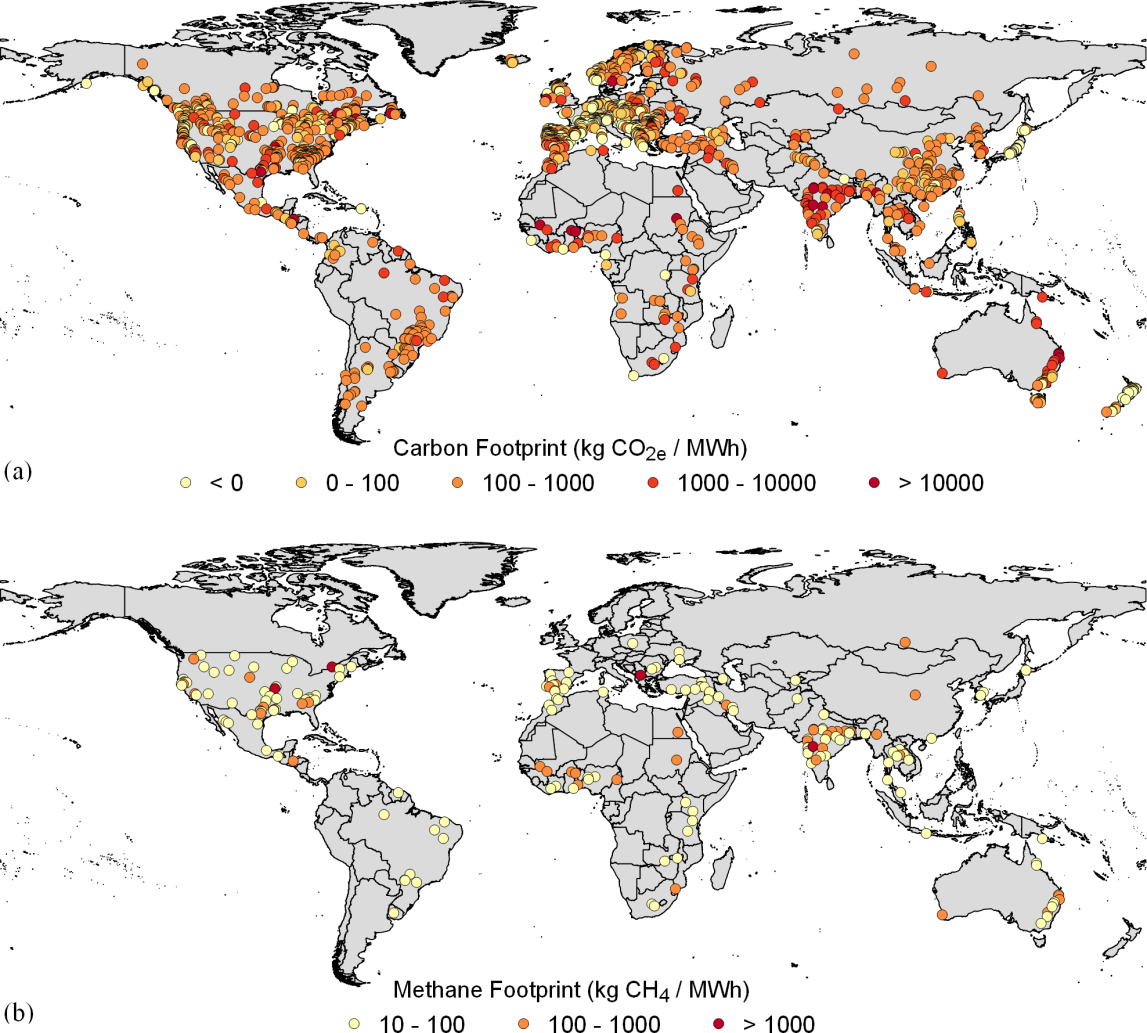
**Carbon footprints of hydropower plants across the world (a) and hydropower plants with high methane emissions (≥ 10 kg CH**_**4**_**/MWh) and a large share of methane emissions (≥ 50% of the carbon footprint) (b).** Country boundaries are obtained from Natural Earth (http://www.naturalearthdata.com/).

We applied the calibrated model to a dataset of 1473 hydropower plants across the world (Fig 1). Our estimate of total carbon emissions exceeds previous estimates by far, due to large CO_2_ emissions. By contrast, our CH_4_ emissions are close to previous estimates. They contribute ~42% to the carbon footprint (Table 2) and therefore do not dominate the footprint results unless a shorter time horizon of 20 years is considered for calculating the GWP. The higher global CO_2_ emissions cannot be explained by outliers, as our maximum value is in the same order of magnitude as the measured maximum (Table 3). Despite the overall higher emission estimates, some plants represent small carbon sinks (Fig 1). Allocation of carbon footprints between multiple purposes of a reservoir leads, on average, to a reduction of ~30% in climate change impacts associated with hydropower (Table 3).

**Table 2:** Global average carbon footprints and methane shares using different approaches.

Model	CO_2e_ (kg/MWh)	Share of CH_4_
Per energy unit (Model 1)	577	16%
Areal fluxes (Model 2)	245	61%
Average of both models (Model A)	411	29%
Corrected average of both models (Model A_C_)	404	42%
Corrected average of both models with GWP20	661	64%

**Table 3:** Global estimates of carbon emissions using the average of both models and applying correction factors without (model A_C_) and with allocation (alloc. A_C_), and results from the training dataset or previous literature (prev.).

		Average (kg/MWh)	Median (kg/MWh)	Max (kg/MWh)	Total (Tg/a)	Total (Tg C/a)
**CO_2_**	Prev.	85.0^a^	74.4^b^	47055^b^	NA	82.0^a^^c^^d^
	Model A_C_	236	102	62733	840	229
	Alloc. A_C_	173	55.1	62733	615	168
**CH_4_**	Prev.	3.5^a^	0.9^b^	2523^b^	NA	7.9^a^^c^^d^
	Model A_C_	4.94	0.63	15072	17.5	13.2
	Alloc. A_C_	2.95	0.43	5024	10.5	7.84
**CO_2e_**	Prev.	NA	NA	NA	288.0^c^	NA
	Model A_C_	404	136	501387	1436	NA
	Alloc. A_C_	273	84.0	167129	970	NA

^a^ Hertwich [8]

^b^ Derived from the training dataset as provided in the supporting information of Barros et al. [7] and Hertwich [8]

^c^ Barros et al. [7]

^d^ The values are recalculated from the average emissions given by Hertwich [8]. The author reported 76 and 7.3 Tg C for CO_2_ and CH_4_, assuming 3288 TWh total hydroelectricity generation in 2009 compared to 3551 TWh assumed in this study. Barros et al. [7] reported 48 and 3 Tg C.

When allocating the impacts between hydropower and other uses, plants with the largest hydroelectricity generation of each continent are below the production-weighted global average emissions, with the exception of the Churchill Falls plant. The high (although not extreme) footprint of the Churchill Fall plant can be attributed partly to the high area-to-electricity ratio and partly to being a single purpose reservoir, not allowing for allocation. Still, each of these largest plants, except Sysenvatnet in Norway, exceed the median (Table 4), indicating that the average footprint is driven by a few hydroelectric power plants with very high emissions.

**Table 4:** Carbon footprints (kg/MWh) of the largest hydropower plant on each continent using the average of the two developed models and applying correction factors without (model A_C_) and with allocation (alloc. A_C_).

Plant	Continent	Electricity (TWh)	Model A_C_	Alloc. A_C_	Share of CH_4_
			CO_2e_	CO_2e_	
Itaipu	SA	91.7	319.5	213.0	7%
Three Gorges	AS	79.9	307.7	153.8	8%
Churchill Falls	NA	30.8	436.4	436.4	5%
Cahora Bassa	AF	15.8	724.2	241.4	54%
Sysenvatnet	EU	4.8	50.0	50.0	<1%
Manapouri	OC	3.3	201.3	201.3	2%

## Discussion

### Climate change impact or renewable energy source?

Although the CH_4_ stocks in tropical reservoirs might contribute to global warming and climate change, they also constitute a potential renewable energy source. The gas can be oxidized in situ or recovered for later energy production with a recovery efficiency of 60% by using low-cost technologies [31]. Using biogenic CH_4_ otherwise emitted to the atmosphere as an energy source is not only cleaner than using the equivalent volume of fossil natural gas, but it also reduces the need for additional hydroelectric reservoirs. This entails far more benefits than reducing the climate change impacts modelled here, as it avoids construction of new reservoirs that lead to resettlements, habitat destruction [31], evaporation and flow alterations [27]. Since the biogeochemical dynamics differ among reservoirs, the potential for climate change mitigation or energy recovery has to be examined case by case [31]. Where energy technologies other than hydropower are more likely to be phased out, different benefits are provided, which might reduce climate change impacts even more.

While on a global level CH_4_ recovery does not seem to be a promising strategy considering the relatively low efficiency of CH_4_ capture combined with a low contribution of CH_4_ to the total carbon footprint of reservoirs, it might be suitable in some locations. We identified 187 dams with a high CH_4_ recovery potential, located mainly in the United States, India and West Africa (Fig 1). If methane emissions from these dams would be captured with the assumed efficiency of 60% [31], 19% of total methane emissions could be saved, which would reduce the overall carbon footprint of global hydropower by 8%.

### Implications

**Fig 2:**
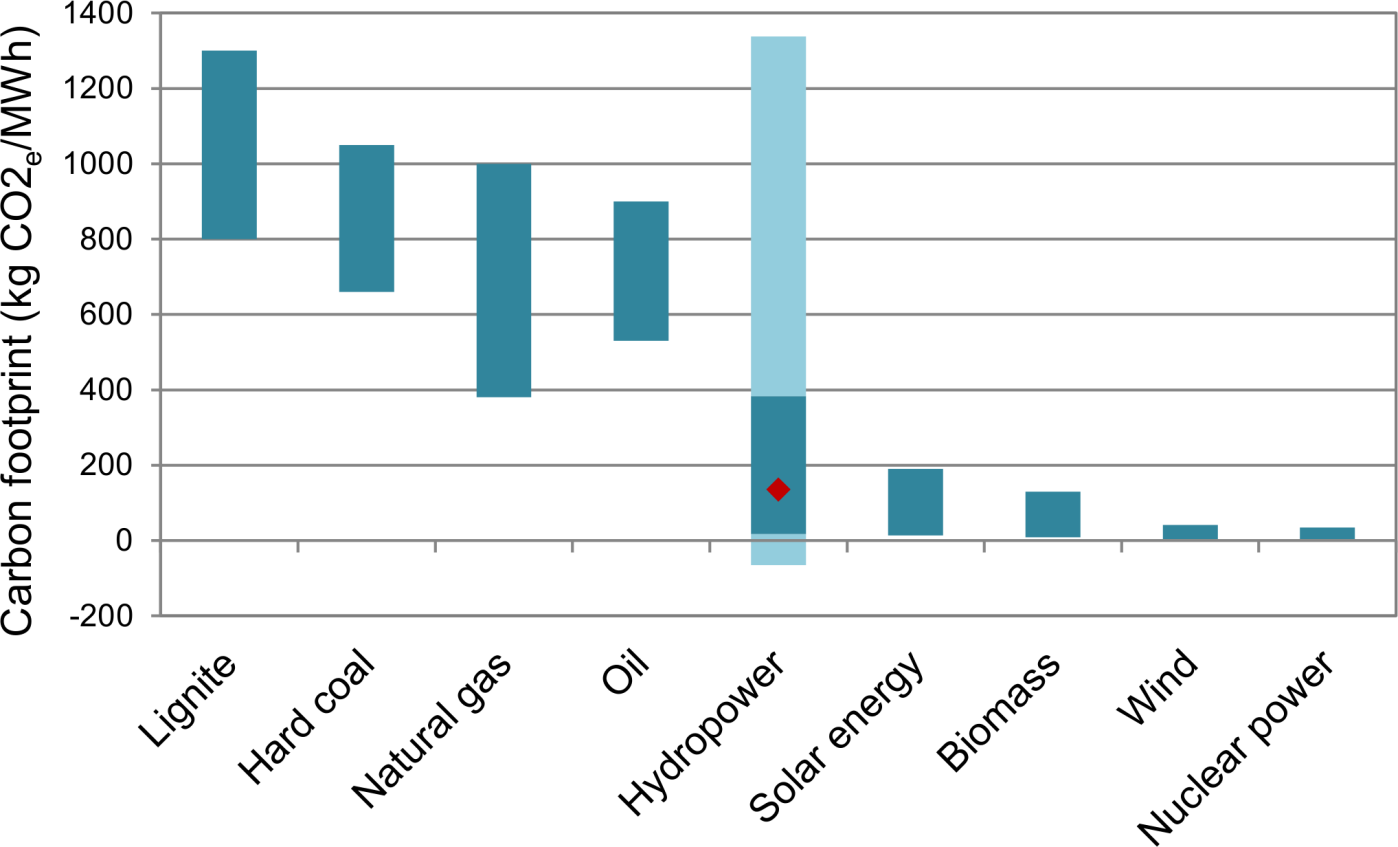
Carbon footprints of various energy sources (based on [32] for all energy sources other than hydropower). The lower and upper value of the dark bar for hydropower are the lower and upper quartiles for the corrected model average (Model A_C_). The light extensions represent the 10 and 90% quantiles and the red diamond marks the median.

Biogenic carbon emissions from hydropower reservoirs are far higher than previously assumed. Consequently, our results question the sustainability that is often associated with hydropower. Although the carbon footprint of hydropower exceeds that of all other renewable energy sources and that of fossil energy sources combined with carbon capture and storage (CSS), it is on average about half the footprint reported for conventional fossil energy sources [32,33] (Fig 2). The emissions vary greatly among plants and the relationship with the reservoir age is not yet well understood, as demonstrated by the contradicting reports addressed above. In addition, uncertainties of estimates remain high, as the comparison of the two approaches (per energy unit and areal fluxes) reveals, with a production-weighted average coefficient of variation (CV) of 57%, 43% and 29% for CO_2_, CH_4_ and CO_2e_ emissions, respectively. This highlights the need for a more extensive monitoring network covering diverse ecosystems, repeated measurements over a longer observation period (at least a decade) as well as standardized measurement procedures taking into account carbon burial [34], drawdown areas [10] and methane bubbles [35]. The dam construction is typically not relevant for the total carbon footprint with emissions of ~19 CO_2e_/MWh [33].

Besides potential environmental damages, reservoirs also have economic and social impacts. Therefore, multiple criteria have to be examined in order to determine the optimal location for a reservoir [30]. Climate change is, although crucial, only one aspect in such a set of criteria. The statistical model set up in this study serves to estimate the carbon footprint of potential new sites prior to reservoir construction. It also identifies those dams where methane emissions are relevant and biogas electricity could be combined with hydropower. Finally, while hydropower has significant GHG emissions, it can also serve as highly efficient energy storage (pumped storage power plant) of alternative energy sources, such as solar or wind power, which can reduce overall emissions if properly combined.

## Supporting Information

S1 File Representation of climate zones in the model training dataset. (PDF)

S2 File Derivation of correction factors (methane ebullition and carbon burial). (PDF)

S3 File Comparison of biomass density in forests and lakes / reservoirs. (PDF)

S1 Table Carbon emissions of hydropower plants and their national and continental averages. (XLSX)
