# Supplementary material for: Hydropower's Biogenic Carbon Footprint
Source: PLoS One. 2016 Sep 14;11(9):e0161947. doi: 10.1371/journal.pone.0161947 (PMC5023102; doi:10.1371/journal.pone.0161947)
Supplement: S1 File — (PDF) [file pone.0161947.s001.pdf]

SUPPORTING INFORMATION

Hydropower's biogenic carbon footprint

Laura Scherer* and Stephan Pfister

Institute of Environmental Engineering, ETH Zurich, 8093 Zurich, Switzerland

*E-mail: scherer@ifu.baug.ethz.ch

S1 Representation of climate zones in the model training dataset

The training dataset contains 154 hydropower plants [1] and covers all five climate zones [2] (**Figure S1**, **Table S1**). The dataset is biased towards Europe, North and South America. China, the biggest hydroelectricity producer worldwide [3], is not included in the dataset; however, most of the hydropower plants in China are situated in the temperate climate zone, which is well represented in the dataset (**Table S1**). In addition, the most important other hydroelectricity producing countries Brazil, Canada and the United States [3] are contained in the dataset (**Figure S1**).

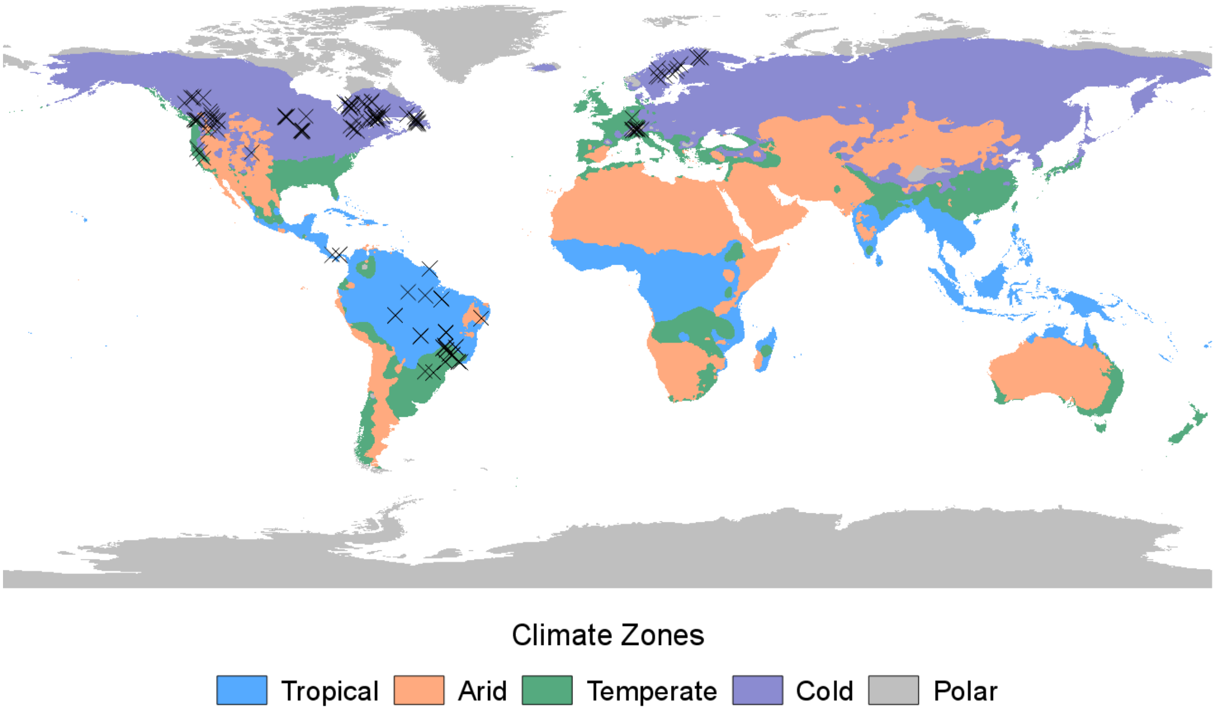

Figure S1. Distribution of hydropower plants used for setting up the model.

Table S1. Representation of climate zones.

Climate zone	Tropical	Arid	Temperate	Cold	Polar
Number	31	2	31	79	9

References

1. Barros N, Cole JJ, Tranvik LJ, Prairie YT, Bastviken D, Huszar VLM, et al. Carbon emission from hydroelectric reservoirs linked to reservoir age and latitude. Nat Geosci. 2011; 4: 593–596. doi: 10.1038/ngeo1211.
2. Peel MC, Finlayson BL, McMahon TA. Updated world map of the Köppen-Geiger climate classification. Hydrol Earth Syst Sci. 2007; 11: 1633–1644. doi: 10.5194/hess-11-1633-2007.
3. IEA. Key world energy statistics. Paris: International Energy Agency; 2014.
