# Supplementary material for: Hydropower's Biogenic Carbon Footprint
Source: PLoS One. 2016 Sep 14;11(9):e0161947. doi: 10.1371/journal.pone.0161947 (PMC5023102; doi:10.1371/journal.pone.0161947)
Supplement: S2 File — (PDF) [file pone.0161947.s002.pdf]

SUPPORTING INFORMATION

Hydropower's biogenic carbon footprint

Laura Scherer* and Stephan Pfister

Institute of Environmental Engineering, ETH Zurich, 8093 Zurich, Switzerland

*E-mail: scherer@ifu.baug.ethz.ch

S2 Derivation of correction factors

Methane ebullition

Almost half of the hydropower plants with methane measurements (42 out 98 → 43%) only considered diffusive fluxes of methane emissions at the reservoir surface, but disregarded the formation of methane bubbles (ebullition) and the emissions at turbines or in rivers downstream of the reservoir. Such emissions can be very high in the tropics and we assumed that they contribute 70% to the total methane emissions of tropical reservoirs based on [1]. According to measurements in US hydroelectric reservoirs, bubbling contributes on average 11% to the total methane emissions [2]; however, measurements often underestimate actual emissions. Comparisons between a novel and a traditional sampling device have demonstrated that the traditional device underestimates methane emissions by a factor of 2 [3]. Assuming that this sampling error is also valid for the US measurements, and the US measurements are representative for temperate or non-tropical reservoirs, bubbling contributes on average ~20% to the total methane emissions of these reservoirs. As a consequence, if bubbling was not considered, methane emissions are 3.3 times the reported value for tropical reservoirs, and 1.2 times the reported value for temperate reservoirs. If bubbling was considered, but is twice as high as reported (based on [3]), methane emissions are 1.5 times the reported value for tropical reservoirs, and 1.1 times the reported value for temperate reservoirs. Considering that bubbling was disregarded for 43% of the methane measurements in the training dataset, and that 21% of the hydropower plants with methane measurements are situated in the tropics, the overall correction factors for the methane emissions predicted with our model is 1.4.

Carbon burial in reservoir sediments

On a global scale, reservoirs are estimated to bury 1466 g CO_2-equivalents m^{-2} a^{-1} [4] and to emit 1533 g CO_2-equivalents m^{-2} a^{-1} [5]. This indicates that reservoirs are emitting slightly more carbon than they bury. Part of the carbon buried in reservoirs would also have been buried in downstream lakes or the ocean [6] so that not all benefits from carbon burial can be attributed to the reservoirs. Based on a 20% lower burial efficiency in lakes than in reservoirs [7] and a 40% lower burial efficiency in oceans than in lakes [6] (i.e. 50% lower than in reservoirs), we assume that without a reservoir still 65% of the carbon burial would occur and only 513 g CO_2-equivalents m^{-2} a^{-1} can be attributed to reservoirs. As a result, only one third of the avoided emissions of a reservoir are buried due to the reservoir (net burial). When assuming that none of the records of the training dataset considers carbon burial, the carbon emissions would have to be reduced on average by 33%, which is an upper estimate. 17 out of 143 hydropower plants with CO_2 measurements have negative CO_2 emissions and, as such, they represent carbon sinks. This shows that carbon burial was considered in parts of the training dataset.

Even if hydropower plants are not sinks, carbon burial might have been considered. In [2], carbon burial was considered, but only 2 out of 6 reservoirs are identified as carbon sinks. Assuming that for double as many plants as carbon sinks in the training dataset carbon burial was considered, but that the efficiency was overestimated by a factor of 3 due to the difference between gross and net burial, then carbon burial is underestimated by about 29%. Overall, we assume that our predicted CO_2-equivalent emissions have to be reduced by 13%.

We acknowledge that these correction factors are rough estimates and entail high uncertainties. Burial efficiencies and the role of methane ebullition vary greatly among plants.

References

1. Fearnside PM, Pueyo S. Greenhouse-gas emissions from tropical dams. Nat Clim Change. 2012; 2: 382–384. doi: 10.1038/nclimate1540.
2. Soumis N, Duchemin É, Canuel R, Lucotte M. Greenhouse gas emissions from reservoirs of the western United States. Glob Biogeochem Cycles. 2004; 18: n/a-n/a. doi: 10.1029/2003GB002197.
3. Kemenes A, Forsberg BR, Melack JM. CO2 emissions from a tropical hydroelectric reservoir (Balbina, Brazil). J Geophys Res. 2011; 116: n/a-n/a. doi: 10.1029/2010JG001465.
4. Dean WE, Gorham E. Magnitude and significance of carbon burial in lakes, reservoirs, and peatlands. Geology. 1998; 26: 535–538. doi: 10.1130/0091-7613(1998)026<0535:MASOCB>2.3.CO;2.
5. Louis VL St., Kelly CA, Duchemin É, Rudd JWM, Rosenberg DM. Reservoir Surfaces as Sources of Greenhouse Gases to the Atmosphere. A Global Estimate. BioScience. 2000; 50: 766–775. doi: 10.1641/0006-3568(2000)050[0766:RSASOG]2.0.CO;2.
6. Sobek S, Durisch-Kaiser E, Zurbrügg R, Wongfun N, Wessels M, Pasche N, et al. Organic carbon burial efficiency in lake sediments controlled by oxygen exposure time and sediment source. Limnol Oceanogr. 2009; 54: 2243.
7. Sobek S, DelSontro T, Wongfun N, Wehrli B. Extreme organic carbon burial fuels intense methane bubbling in a temperate reservoir. Geophys Res Lett. 2012; 39: L01401. doi: 10.1029/2011GL050144.
