# Supplementary material for: Hydropower's Biogenic Carbon Footprint
Source: PLoS One. 2016 Sep 14;11(9):e0161947. doi: 10.1371/journal.pone.0161947 (PMC5023102; doi:10.1371/journal.pone.0161947)
Supplement: S3 File — (PDF) [file pone.0161947.s003.pdf]

SUPPORTING INFORMATION

Hydropower's biogenic carbon footprint

Laura Scherer* and Stephan Pfister

Institute of Environmental Engineering, ETH Zurich, 8093 Zurich, Switzerland

*E-mail: scherer@ifu.baug.ethz.ch

S3 Comparison of biomass density in forests and lakes / reservoirs

We compare the biomass densities in forests and lakes (as proxy for reservoirs) in order to support the claim that potential sequestration of CO_2 in algae and fish biomass of dams is marginal compared to terrestrial vegetation. Temperate forests have a biomass density of ~13 kg/m^2, assuming a shoot biomass of 10 kg/m^2 and a root-to-shoot ratio of 0.3 [1]. By contrast, temperate lakes or reservoirs have a biomass density of 0.21 kg/m^2, assuming an average algal density of 3 g/m^3 [2] and an average depth of 70 m, which is slightly higher than the average dam height in our database. This result is therefore generally over-estimating algal biomass in reservoirs. Consequently, the ratio of biomass density in forests compared to lakes/reservoirs amounts to ~62. CO_2 sequestration in reservoirs can therefore be considered marginal compared to previous vegetation (<2%), considering overall uncertainty of the model.

References

1. Mokany K, Raison RJ, Prokushkin AS. Critical analysis of root : shoot ratios in terrestrial biomes. Glob Chang Biol. 2006; 12: 84–96. doi: 10.1111/j.1365-2486.2005.001043.x.
2. Beaulieu M, Pick F, Palmer M, Watson S, Winter J, Zurawell R, et al. Comparing predictive cyanobacterial models from temperate regions. Can J Fish Aquat Sci. 2014; 71: 1830–1839. doi: 10.1139/cjfas-2014-0168.
